# Exploring Predictors of Genetic Counseling and Testing for Hereditary Breast and Ovarian Cancer: Findings from the 2015 U.S. National Health Interview Survey

**DOI:** 10.3390/jpm9020026

**Published:** 2019-05-10

**Authors:** Caitlin G. Allen, Megan Roberts, Yue Guan

**Affiliations:** 1 Rollins School of Public Health, Emory University, 1518 Clifton Road NE, Atlanta, GA 30307, USA; yue.guan@emory.edu; 2 Division of Cancer Control and Population Sciences, National Cancer Institute, 9609 Medical Center Drive, Rockville, MD 20850, USA; megan.roberts@unc.edu

**Keywords:** genetics, hereditary cancer, HBOC, *BRCA1/2*, genetic counseling, genetic testing, genetic services

## Abstract

Despite efforts to increase the availability of clinical genetic testing and counseling for Hereditary Breast and Ovarian (HBOC)-related cancers, these services remain underutilized in clinical settings. There have been few efforts to understand the public’s use of cancer genetic services, particularly for HBOC-related cancers. This analysis is based on data from the 2015 National Health Interview Survey (NHIS), a U.S.-based nationwide probability sample, to better understand the public’s use of HBOC-related clinical cancer genetic services. Bivariate analyses were used to compute percentages and examine the associations of familial cancer risk for three genetic services outcomes (ever had genetic counseling for cancer risk, ever discussed genetic testing for cancer risk with a provider, and ever had genetic testing for cancer risk). Multivariable logistic regression models were used to estimate the association of familial cancer risk and other demographic and health variables with genetic services. Most women (87.67%) in this study were at low risk based on self-reported family history of breast and ovarian cancer, 10.65% were at medium risk, and 1.68% were at high risk. Overall, very small numbers of individuals had ever had genetic counseling (2.78%), discussed genetic testing with their physician (4.55%) or had genetic testing (1.64%). Across all genetic services outcomes, individuals who were at higher familial risk were more likely to have had genetic counseling than those at lower risk (high risk: aOR = 5.869, 95% CI = 2.911–11.835; medium risk: aOR = 4.121, 95% CI = 2.934–5.789), discussed genetic testing (high risk: aOR = 5.133, 95% CI = 2.699–9.764; medium risk: aOR = 3.649, 95% CI = 2.696–4.938), and completed genetic testing (high risk: aOR = 8.531, 95% CI = 3.666–19.851; medium risk aOR = 3.057, 95% CI = 1.835–5.094). Those who perceived themselves as being more likely to develop cancer than the average woman were more likely to engage in genetic counseling (aOR = 1.916, 95% CI = 1.334–2.752), discuss genetic testing (aOR = 3.314, 95% CI = 2.463–4.459) or have had genetic testing (aOR = 1.947, 95% CI = 1.13–3.54). Personal cancer history was also a significant predictor of likelihood to have engaged in genetic services. Our findings highlight: (1) potential under-utilization of cancer genetic services among high risk populations in the U.S. and (2) differences in genetic services use based on individual’s characteristics such as self-reported familial risk, personal history, and beliefs about risk of cancer. These results align with other studies which have noted that awareness and use of genetic services are low in the general population and likely not reaching individuals who could benefit most from screening for inherited cancers. Efforts to promote public awareness of familial cancer risk may lead to better uptake of cancer genetic services.

## 1. Introduction

Women who carry a *BRCA1/2* pathogenic variant have substantially increased lifetime risks for breast and ovarian cancer compared to the general population [1]. The identification of pathogenic variant carriers is a precursor to the use of tailored management and prevention strategies to reduce mortality in individuals and to initiate testing among at-risk family members. Further, confirmation of true negatives could reduce unnecessary cancer screening and surgery [2,3,4,5]. In response to this evidence, several medical associations have put forth clinical practice guidelines to promote screening for hereditary breast and ovarian cancer (HBOC), and as appropriate, genetic counseling and testing [2,6,7,8,9,10,11]. 

Despite well-defined strategies for screening among individuals who are at increased risk for *BRCA1/2*-related cancers, cancer genetic services remain underutilized in clinical settings [12,13,14,15,16]. The vast majority of individuals who carry *BRCA1/2* pathogenic variant have not yet been identified, and when they are, it is often within the context of a breast and/or ovarian cancer diagnosis [17]. Genetic counseling referrals and genetic testing rates are low even among individuals with cancer diagnoses. Indeed, half or fewer of the breast cancer and ovarian cancer patients have received genetic counseling or testing [18,19,20,21], and many have never discussed genetic testing with their provider [22]. 

Since 2005, the U.S. Preventive Services Task Force has recommended screening for unaffected (i.e., no personal history of cancer) women with a strong family history of certain cancers to identify those who may be at increased risk for potentially harmful pathogenic variants in *BRCA1/2* [23]. It is recommended that these higher risk women be referred for genetic counseling and undergo genetic testing if indicated after counseling, while those deemed at low inherited cancer risk should not be recommended for routine genetic counseling or testing [10]. Prior analyses of national data found that there was low use of genetic services among individuals at risk of *BRCA1/2*- or Lynch syndrome-associated cancers; however, since 2005, there have been substantial legal and social changes that would likely influence genetic service use (e.g., decrease in test cost, better insurance coverage, Genetic Information Nondiscrimination Act) [24]. More recent studies have reported very low uptake rates of genetic counseling and testing among individuals at-risk for a *BRCA1/2* pathogenic variant [25] and the status of misuse of genetic services among low risk women is largely unknown. Although there have been numerous efforts undertaken to improve public awareness about familial risk, health history and genetic testing to help increase the population’s genetic literacy [26,27,28,29], there are still substantial gaps in uptake of recommendations and current patterns in use of genetic services remain unclear [26,27,28,29]. Additional attention has also been drawn to this issue due to the increase in access to direct-to-consumer testing that provides genomic profiling to the public [30,31,32,33,34,35]. 

Therefore, the goal of this paper is to use the updated 2015 U.S. National Health Information Survey (NHIS) data to identify the likelihood that individuals have had genetic counseling, discussed genetic testing, or had genetic testing based on key demographic variables and familial risk of *BRCA1/2*-related cancers. 

## 2. Methods

This secondary data analysis is based on data from the 2015 National Health Interview Survey (NHIS), which is a national probability sample survey that collects information about the United States population through annual household interviews [36]. Due to the nature of this study (publicly available data, non-human subjects research), no IRB approval was required. The sampling plan was designed to gather information about clusters of addresses located in primary sampling units drawn from each state and the District of Columbia. The Cancer Control Supplement is distributed every five years and includes a set of questions assessing personal and family history of cancer and knowledge and use of genetic testing, among other health-related questions. The population for the present study was restricted to adult females who provided a response to at least one of the three outcome variable questions (ever had genetic counseling, discussed genetic testing, had genetic testing). 

***Outcome Variables***. Three outcome variables pertaining to genetic testing and counseling for cancer were included from the Cancer Control module. Participants were provided with a definition of genetic testing and counseling and were asked if they have ever had genetic counseling by responding to the question, “Have you ever received genetic counseling for cancer risk” (yes/no), ever discussed genetic testing for cancer by responding to the question, “Have you ever discussed the possibility of getting a genetic test for cancer risk with a doctor or other health care professional,” (yes/no), or ever had genetic testing for cancer by responding to the question, “Have you ever had a genetic test to determine if you are at greater risk of developing cancer in the future” (yes/no). Individuals only received the final question about whether they ever had genetic testing if they responded ‘yes’ to the previous question about ever discussing genetic testing for cancer with a provider. 

***Independent Variables.*** Individual’s familial risk for *BRCA1/2*-related cancers (breast and ovarian) was estimated based on their reported number of first-degree female relatives (parents, siblings, offspring) who had been diagnosed with *BRCA1/2*-related cancers (i.e., breast and ovarian cancers) and the age of diagnosis (<50, ≥50 years of age). Familial risk was ranked as (1) “low risk” which included individuals with no first degree relatives with a history of breast or ovarian cancers, (2) “medium risk” that included women with at least one first-degree relative diagnosed with breast cancer at ≥50 years of age, or (3) “high risk” that included at least one first-degree relative diagnosed with breast cancer under the age of 50 and/or any family history of ovarian cancer [37]. These classifications were adapted from previous studies that have used NHIS data [24]. Additional variables of interest included age (18+ years), race/ethnicity (non-Hispanic White, non-Hispanic Black, and Other), marital status (married, widowed, separated/divorced, never married, living with partner), highest level of education (0–21), household income ($0–$34,999, $35,000–$49,000, $50,000–$74,999, $75,000–$99,999, and $100,000 and over), type of insurance coverage (Private, Other), individual’s perception about their likelihood to get breast cancer compared to the average woman (less likely, about as likely, more likely), and personal cancer history (none, breast or ovary, other cancer). 

***Statistical Analysis.*** We conducted a complete case analysis using the sample of women who answered questions about genetic testing and counseling. Women who responded to at least one of these three questions were included in the sample.

We used weighted bivariate analyses to compute percentages and examine the associations of familial cancer risk with other characteristics and with each of the genetic testing and counseling outcomes. Weighted two-sample t-test (for age and education) and chi-square tests of independence (for categorical variables) were used to determine statistical significance for each outcome. 

Multivariable logistic regression models were used to estimate adjusted odds ratios and corresponding 95% confident intervals for the association of familial cancer risk and covariates with each of the genetic testing and counseling variables. We controlled for all factors that were determined a priori to potentially affect genetic testing outcomes based on previous literature and model fit [38]. Thus, the final model included: level of familial risk, age, race/ethnicity, marital status, highest level of education, household income, insurance status, perception about an individual’s likelihood to get cancer, and personal cancer history. All analyses were conducted in SAS version 9.4 and incorporated the survey sample weights to account for the sampling strategy, non-response, and design effect of cluster sampling in NHIS.

## 3. Results

A total of 18,601 women were included in this nationally representative sample. Most (87.67%) were at low risk (no first-degree female relatives with history of breast or ovarian cancer), 10.65% were at medium risk (at least one first-degree female relative with breast cancer), and 1.68% were at high risk (at least one first-degree relative diagnosed with breast cancer under 50 or any first-degree relatives with ovarian cancer) of developing *BRCA1/2*-related cancers. Overall, very small numbers of individuals had ever had genetic counseling (2.78%), discussed genetic testing with their physician (4.55%) or had genetic testing (1.64%) (Table 1). 

Predictors of likelihood to have ever had genetic counseling (Table 2) included level of familial risk, with those individuals with the highest family risk (aOR = 5.869, 95% CI = 2.911–11.835) and those with medium family risk (aOR = 4.121, 95% CI = 2.934–5.789) to be more likely to have had genetic counseling than those at low risk. Other factors associated with genetic counseling included perceived cancer risk. Individuals who felt they were about as likely to develop cancer were significantly more likely to have received genetic counseling compared to those that perceived their risk to be less likely than their peers (aOR = 1.916, 95% CI = 1.334–2.752). Personal history of breast or ovarian cancer also increased likelihood to have had genetic counseling compared to those who had no personal history of cancer (aOR = 11.814, 95% CI = 7.236–19.291) and other cancer (aOR = 3.317, 95% CI = 2.003–5.491).

Level of familial risk also influenced likelihood to have discussed genetic testing (Table 3), with those at medium (aOR = 3.649, 95% CI = 2.696–4.938) and high risk (aOR = 5.133, 95% CI = 2.699–9.764) being more likely than those who were at low risk to have ever discussed genetic testing with a provider. In addition, those who perceived themselves as more likely to develop cancer (aOR = 3.314, 95% CI = 2.463–4.459) were more likely to have discussed genetic testing than individuals who perceived themselves to be as likely as their peers to develop cancer. Individuals with a personal history of breast or ovarian cancer were more likely to have discussed genetic testing compared to those with no personal history of cancer (aOR = 8.473, 95% CI = 5.224–13.744) and other cancer (aOR = 2.612, 95% CI = 1.693–4.029). 

The final outcome of whether an individual has had genetic testing for cancer risk (Table 4) was associated with individual’s level of risk, with those who are at medium or high risk being significantly more likely to have ever had genetic testing than those at lowest risk (medium aOR = 3.057, 95% CI = 1.835–5.094; high aOR = 8.531, 95% CI = 3.666–19.851). Perceived cancer risk was also significantly associated with likelihood to have had genetic testing for cancer risk with those who perceived themselves to be at higher risk to develop cancer having a higher likelihood to have had genetic testing (aOR = 1.947, 95% CI = 1.13–3.354). Personal cancer history was also associated with higher likelihood to have had genetic testing (breast or ovary aOR = 20.266, 95% CI = 11.122–36.927; other cancer aOR = 3.777, 95% CI = 2.052–6.952).

## 4. Discussion

Despite efforts to increase utilization of genetic services among individuals at risk for developing *BRCA1/2-*related cancers, our findings show potential underutilization in this nationally representative sample of females in the U.S. Higher levels of familial risk were associated with higher levels of genetic counseling, discussion of testing, and use of genetic testing; however, overall levels of engagement with genetic services were low. While over 12% of participants would have been considered eligible for genetic counseling and subsequent genetic testing based on family history, only a small subset received these services, suggesting that individuals at increased likelihood of HBOC are potentially not receiving appropriate follow-up services. These results align with previous reports that demonstrate low rates of awareness and utilization of counseling and testing, even among individuals at high risk of hereditary cancer [24,39,40] and with personal history of cancer [22]. It has been estimated that only 6% of *BRCA1/2* mutation carriers in the general population have been identified and a recent study found fewer than one-in-five at-risk breast or ovarian cancer patients have undergone genetic testing [41]. 

We found significant differences across all genetic services outcomes based on individual’s perceived cancer risk. Individuals with higher risk perceptions may be more likely to engage with health professionals and be willing to discuss counseling and testing compared to those who are less worried [42]. Indeed, cancer risk and worry are well established motivators for preventive behaviors [43,44,45,46]. Future efforts to improve genetic service use could consider tailoring interventions based on perceived cancer risk. 

Our findings also suggest an association between personal cancer history and likelihood to engage in genetic services. This increased genetic service utilization among individuals with a personal history of cancer indicates a linkage between genetic service use and cancer diagnosis [17]. While these findings point to possible integration of genetic services into clinical practice, they do not provide insight about the number of women who were diagnosed and did *not* receive appropriate follow-up. Recent guidelines have suggested moving toward multigene panel testing for all breast cancer patients [47,48]. This approach could help identify more patients with hereditary cancers; approximately 9% of patients with breast cancer who had tested negative for *BRCA1/2* mutations and underwent subsequent panel testing were found to have a pathogenic mutation in a breast cancer susceptibility gene [49]. Furthermore, future studies should continue to monitor use of these health services among individuals diagnosed with HBOC and consider ways to improve cascade screening among family members who may also be at elevated risk. 

Some of our results deviated from the literature, as we did not find significant differences in uptake of genetic counseling or genetic testing by race. Other studies have identified low levels of awareness and use of genetic counseling and testing among minority individuals. Efforts to expand genetic service use come in the context of widespread concerns that health benefits of genomic translation will not reach all those who could benefit and thus, will exacerbate health disparities [50,51,52,53]. Indeed, uptake of evidence-based recommendations for genetic services already has shown patterns of disparities for African American women even in specialty care settings [14,15]. These deviant findings may have been due to power limitations, as we had limited cell sizes when including all predictors of interest in the final model. In addition, these null results may be due to the self-reported nature of these data. Previous literature has demonstrated that family health history collection and genetic literacy are lower among minority groups, which could reduce the accuracy of recalling health information about family members [54,55,56,57].

While our results have important implications, this study is not without limitations. The Cancer Control Supplemental survey used for this analysis focused on *BRCA1/2*-related cancers among women; however, *BRCA1/2* mutations can also occur in men and recent results have demonstrated a gender gap in genetic testing [58]. Additionally, other hereditary cancers (e.g., Lynch Syndrome-associated cancers) with known genetic tests were not assessed. We assessed individual’s likelihood to have ever received cancer genetic services and thus did not directly assess whether an individual had received services specific to HBOC. Thus, it is possible that those who received genetic services specifically for the purposes of HBOC or *BRCA1/2* variants is even lower than what is reported in our results. Further, these data are cross sectional and self-reported, thus, we are unable to assess for causality and whether cancer risk predicted or preceded genetic counseling and testing uptake. The self-reported nature of data may limit findings, as these individuals may not know their full health history and there is opportunity for confusion about cancer types, especially among ovarian cancer, which may be mistaken for cervical and uterine cancer. In addition, the classification we used for familial cancer risk (low, medium, and high) was based on self-reported family history for *BRCA1/2*-related cancers. This approach was adapted from previous studies that have used NHIS [24]. However, the NHIS survey questions were limited in their ability to evaluate family history that may be suggestive of other high penetrance breast and colon cancer genes (e.g., *TP53, PTEN*) and moderate penetrance genes (e.g., *ATM, CHEK2*) [59,60]. Given that the multigene panel testing is becoming the standard clinical practice, it is important to include more detailed family cancer history questions in national surveys to estimate familial risk for hereditary cancers. Finally, our estimates of risk focused on family history risk factors, rather than personal risk factors (e.g., smoking, alcohol consumption, age at menarche, age at first live birth of child). 

In light of these limitations, our results have important implications at both the clinical and public health levels. Despite ongoing efforts to increase family health history knowledge and collection of family history at the individual and provider levels to improve risk stratification and referrals to counseling and testing, this study demonstrates that individuals at high risk may not be receiving appropriate information and referrals to genetic services. There is well-documented evidence indicating a lack of time spent on discussion of family health history and a wide range of variation in physician practice around collection of health histories [61,62]. Further, patients do not typically collect or report family health information to physicians and may avoid genetic counseling and testing because of privacy concerns and fear of adverse consequences, including costs and life insurance discrimination [63,64,65]. Even if health histories are properly collected, providers often report insufficient knowledge of genetics and lack of resources for referral to genetic services (e.g., do not know about hospital cancer genetic services), with concern about shortage of adult medical genetic counselors [66,67,68,69,70,71,72,73,74]. Widespread efforts could be supported and sustained by improving baseline family health history collection through easy-to-use tools and provider decision support [32,75]. Improving collection of health history information for both individuals and providers could improve prevention efforts for individuals at risk of *BRCA1/2*-related cancers. 

Telegenetic counseling service also holds the promise to expand cancer genetic services reach, especially among racial-ethnic minority groups, people in rural settings and with lower socioeconomic status [76,77]. The implementation of new testing criteria to offer panel testing to all breast cancer patients could potentially maximize the identification of hereditary cancer patients and promote the intervention efficacy for patients and their at-risk family members. Other strategies to increase knowledge and use of genetic services may include bidirectional cancer registry reporting provider and patient education about cancer genetics [78,79]. Use of large datasets also will help ensure we are working toward addressing broad national goals (e.g., Healthy People 2020 genomics goals) [80]. Specifically, use of the NHIS and other datasets can help to identify potential characteristics of individuals who are and are not accessing genetic services appropriately and thus target interventions to meet this goal in the future.

Findings of this study could inform future research and intervention elements such as strategies to improve collection of health history information, and ultimately improve appropriate referral and use of cancer genetic services for the benefit of all patients and their families. 

## Figures and Tables

**Table 1:** Characteristics of Individuals by Knowledge and Use of Genetic Counseling and Testing for Cancer.

	Ever Had Genetic Counseling (*N* = 475)		Discussed Genetic Testing (*N* = 778)		Ever had Genetic Testing (*N* = 280)	
	*N* (Mean)	% (95% CI)	*N* (Mean)	% (95% CI)	*N* (Mean)	% (95% CI)
**Level of Familial Risk**						
Low	285	61.624	482	61.208	168	61.747
Medium	162	33.795	261	34.987	89	30.382
High	25	4.581	33	3.805	23	7.871
**Age**	52.23	50.35–54.11	49.10	47.58–50.63	53.39	51.48–55.29
**Race/Ethnicity**						
Non-Hispanic White	315	71.476	527	72.953	188	72.525
Non-Hispanic Black	78	14.988	111	13.629	44	13.712
Other	82	13.536	140	13.417	48	13.764
**Marital Status**						
Married	215	48.972	345	46.934	128	49.218
Widowed	54	11.544	64	9.406	31	11.501
Separated/Divorced	109	20.65	152	15.686	58	18.956
Never Married	72	14.399	170	22.203	49	15.381
Living with Partner	25	4.434	47	5.771	14	4.947
**Highest Level of Education**	15.85	15.49–16.21	16.25	15.99–16.52	15.79	15.41–16.16
**Household Income**						
$0–34,999	158	33.213	253	31.988	86	32.862
$35,000–$49,000	49	9.872	82	10.305	36	11.504
$50,000–$74,999	79	18.559	118	16.774	51	20.267
$75,000–$99,999	44	9.747	79	10.808	21	6.634
$100,000 and over	119	28.608	190	30.125	72	28.732
**Insurance Status**						
Private (ref)	244	73.379	448	77.138	148	71.358
Other	115	26.620	176	22.862	67	28.643
**Perceived Cancer Risk in Self**						
More Likely	165	38.359	267	34.422	94	34.769
Less Likely	121	26.858	179	23.864	74	29.914
About as Likely	172	34.783	311	41.714	101	35.317
**Personal Cancer History**						
No cancer	315	65.08	572	71.782	155	55.857
Breast of Ovarian	97	20.27	111	15.647	82	27.635
Other cancer	63	14.65	95	12.571	43	16.509

Weighted percent are reported.

**Table 2:** Associations with Ever had Genetic Counseling.

	OR	95% CI		aOR	95% CI	
**Level of Familial Risk**						
Low (ref)						
Medium	4.863 *	3.885	6.089	4.121 *	2.934	5.789
High	4.102 *	2.350	7.160	5.869 *	2.911	11.835
**Age**	0.983	0.964	1.001	0.991	0.978	1.005
**Race/Ethnicity**						
Non-Hispanic White (ref)						
Non-Hispanic Black	1.073	0.780	1.475	1.532	0.984	2.384
Other	0.685 *	0.516	0.909	0.969	0.696	1.348
**Highest Level of Education**	1.067	1.019	1.117	1.016	0.946	1.091
**Marital Status**						
Married (ref)						
Widowed	0.718	0.488	1.055	0.721	0.247	2.1
Separated/Divorced	0.957	0.718	1.275	0.982	0.671	1.435
Never Married	0.591 *	0.426	0.820	0.751	0.483	1.169
Living with Partner	0.654	0.369	1.159	0.655	0.344	1.248
**Household Income**						
$0–34,999	0.543 *	0.386	0.764	0.693	0.395	1.217
$35,000–$49,000	0.570 *	0.380	0.855	0.732	0.427	1.254
$50,000–$74,999	0.822	0.576	1.173	0.826	0.523	1.305
$75,000–$99,999	0.628	0.407	0.968	0.807	0.487	1.336
$100,000 and over (ref)						
**Insurance Status**						
Private (ref)						
Other	0.766	0.578	1.014	0.862	0.564	1.319
**Perceived Cancer Risk in Self**						
Less Likely	0.830	0.627	1.098	0.853	0.604	1.204
About as Likely (ref)						
More Likely	3.885 *	2.991	5.046	1.916 *	1.334	2.752
**Personal Cancer History**						
No Cancer (ref)						
Breast or Ovary	9.721 *	7.139	13.235	11.814 *	7.236	19.291
Other Cancer	2.887 *	2.008	4.152	3.317 *	2.003	5.491

*p < 0.05.

**Table 3:** Associations with Discussed Genetic Testing.

	OR	95% CI		aOR	95% CI	
**Level of Familial Risk**						
Low (ref)						
Medium	5.335 *	4.432	6.422	3.649 *	2.696	4.938
High	3.481 *	2.174	5.572	5.133 *	2.699	9.764
**Age**	0.996	0.992	1.001	0.988 *	0.977	0.998
**Race/Ethnicity**						
Non-Hispanic White (ref)						
Non-Hispanic Black	0.953	0.754	1.205	1.285	0.915	1.805
Other	0.661 *	0.522	0.838	0.892	0.655	1.216
**Highest Level of Education**	1.131	1.087	1.178	1.096	1.029	1.167
**Marital Status**						
Married (ref)						
Widowed	0.604 *	0.444	0.823	1.066	0.449	2.529
Separated/Divorced	0.752 *	0.576	0.981	0.729	0.518	1.025
Never Married	0.961	0.748	1.235	1.197	0.844	1.698
Living with Partner	0.894	0.642	1.247	0.861	0.556	1.334
**Household Income**						
$0–34,999	0.490 *	0.387	0.620	0.743	0.516	1.072
$35,000–$49,000	0.559	0.400	0.781	0.775	0.512	1.175
$50,000–$74,999	0.696 *	0.527	0.920	0.651 *	0.448	0.945
$75,000–$99,999	0.657 *	0.448	0.961	0.777	0.528	1.144
$100,000 and over (ref)						
**Insurance Status**						
Private (ref)						
Other Coverage	0.617	0.494	0.771	0.811	0.596	1.103
**Perceived Cancer Risk in Self**						
Less Likely	0.820	0.651	1.034	0.806	0.599	1.084
About as Likely (ref)						
More Likely	5.600	4.504	6.963	3.314 *	2.463	4.459
**Personal Cancer History**						
No Cancer						
Breast or Ovary	7.048 *	5.360	13.038	8.473 *	5.224	13.744
Other Cancer	2.261 *	1.683	3.038	2.612 *	1.693	4.029

*p < 0.05.

**Table 4:** Associations with Genetic Testing for Cancer Risk.

	OR	95% CI		aOR	95% CI	
**Level of Familial Risk**						
Low (ref)						
Medium	4.200 *	3.081	5.725	3.057 *	1.835	5.094
High	7.083 *	3.851	13.025	8.531 *	3.666	19.851
**Age**	1.009 *	1.002	1.015	0.993	0.976	1.011
**Race/Ethnicity**						
Non-Hispanic White (ref)						
Non-Hispanic Black	0.966	0.652	1.432	1.291	0.742	2.246
Other	0.690	0.468	1.016	0.866	0.526	1.426
**Highest Level of Education**	1.058	1.004	1.115	1.01	0.935	1.091
**Marital Status**						
Married (ref)						
Widowed	0.715	0.437	1.169	0.753	0.244	2.321
Separated/Divorced	0.875	0.575	1.330	0.864	0.506	1.477
Never Married	0.633*	0.413	0.971	0.79	0.409	1.526
Living with Partner	0.731	0.402	1.332	0.639	0.245	1.664
**Household Income**						
$0–34,999	0.541 *	0.365	0.802	0.726	0.386	1.365
$35,000–$49,000	0.670	0.416	1.081	0.996	0.539	1.838
$50,000–$74,999	0.898	0.571	1.411	1.023	0.588	1.78
$75,000–$99,999	0.427 *	0.229	0.796	0.431 *	0.193	0.96
$100,000 and over (ref)						
**Insurance Status**						
Private (ref)						
Other Coverage	0.852	0.590	1.230	0.997	0.603	1.65
**Perceived Cancer Risk in Self**						
Less Likely	1.023	0.710	1.475	1.136	0.722	1.786
About as Likely (ref)						
More Likely	4.232 *	2.985	5.999	1.947 *	1.13	3.354
**Personal Cancer History**						
No Cancer						
Breast or Ovary	14.960 *	10.295	21.738	20.266 *	11.122	36.927
Other Cancer	3.749 *	2.443	5.754	3.777 *	2.052	6.952

* *p* < 0.05.
